# ASH ISTH NHF WFH 2021血管性血友病诊断指南解读

**DOI:** 10.3760/cma.j.issn.0253-2727.2021.05.002

**Published:** 2021-05

**Authors:** 峰 薛, 仁池 杨

**Affiliations:** 中国医学科学院血液病医院（中国医学科学院血液学研究所），实验血液学国家重点实验室，国家血液系统疾病临床医学研究中心，天津 300020 Insitute of Hematology and Blood Diseases Hospital, Chinese Academy of Medical Sciences, State Key Laboratory of Experimental Hematology, National Clinical Research Center for Blood Diseases, Tianjin 300020, China

血管性血友病（VWD）是一种由于血管性血友病因子（VWF）异常所导致的遗传性出血性疾病。VWF在血液循环中结合并稳定凝血因子Ⅷ（FⅧ），并且在血小板黏附及聚集过程中发挥关键作用[1]。VWD的出血特点主要为皮肤黏膜出血，如瘀斑、鼻衄、口腔出血、月经过多、胃肠道出血、牙科治疗后出血、分娩及手术时出血等，严重患者可出现与血友病相同的肌肉及关节出血[1]。不同患者出血的严重程度有所不同，同一患者的出血表现也可能随着时间的推移而变化。

VWD可以分为1型、2型及3型，其中1型及3型分别为VWF部分及完全缺乏，2型为VWF功能缺陷，包括2A、2B、2N、2M型[1]。不同亚型以及不同临床表型导致VWD的诊断和治疗十分复杂。在临床实践中，VWD患者面临的主要问题是不能得到及时准确的诊断，其主要障碍包括缺乏识别异常出血的能力、无法确定合适的诊断方法、专业实验室检测能力有限[2]。

随着新检测方法的不断出现及更多循证医学证据的更新，一些在临床诊疗过程中尚无确切结论或新出现的问题亟需阐述，例如新的VWF血小板依赖性活性检测、1C型VWD的诊断。因此，美国血液学会（ASH）、国际止血与血栓学会（ISTH）、美国国家血友病基金会（NHF）、世界血友病联盟（WFH）联合制定了2021年版VWD诊断指南（下文简称2021指南）[3]。笔者将对2021指南的制定流程、临床问题及推荐等方面做出解读，鉴于指南中某些临床场景或者实验室检查可及性在中国有所不同，笔者也将结合中国实践点评。

一、2021指南基本信息

1. 目标、受众及应用：2021指南的目标是为VWD的诊断提供循证建议，为制定共同决策提供支持。提高受累患者的正确诊断率、减少不必要的检验及避免过度诊断的危害，最终将改善针对患者的宣传教育、有效治疗出血事件以及预防出血。

指南的目标受众包括血液科医师、全科医师、内科医师、其他临床医师、患者以及为改善VWD患者生活而制定地区/国家政策的决策者。指南将帮助临床医师作出合适的诊断治疗决策，也可以为政策、教育和宣传提供信息，并阐述进一步研究需要。但是临床医师在作出决策时必须基于每例患者的实际临床表现，并且考虑患者的价值观和对所选方案预期结果的偏好。需要指出的是，指南并不能覆盖所有临床实践场景，且随着证据的不断更新及新证据的出现，指南的内容也可能会过时而需要更新，另外，遵循指南并不一定保证良好的效果。

2. 指南制定组织、资助及利益冲突：该指南由ASH、ISTH、NHF、WFH四个组织按照ASH相关政策以及G-I-N-McMaster指南制定程序的指导联合制定，并接受ASH指南监督小组委员会的监督。

指南制定专家组成员由四个合作组织提名并由ASH审查及任命。最终纳入了具有该领域临床及研究专业知识的儿科及成人血液学家、内科医师、实验室专家，此外还有4名患者代表。

指南制定由ASH、ISTH、NHF及WFH资助。ASH工作人员及ASH指南监督小组委员会审查了专家组成员披露的信息，确保包含具有不同专业知识和观点的专家，同时避免多数人有相同或类似的冲突。在指南推荐审议过程中，每一条推荐中存在利益冲突的成员可以参与讨论但没有投票资格。

3. 临床问题的确定及临床推荐的制定：在指南制定专家组成立前，首先成立了一个由临床医师，患者，ASH、ISTH、NHF、WFH等的代表组成工作组，制定了初始的临床问题并通过对利益攸关者（医师、患者及看护者）国际性调查进行优先级排序，最终形成了10个能够影响患者结局的问题，作为指南制定的框架[4]。

堪萨斯大学医学中心（KUMC）的“结局及实施小组”建立方法学团队，利用GRADEpro指南开发工具准备了“证据到决策”（EtD）框架材料，针对每个问题进行系统性文献评价总结于EtD表格，并在指南制定专家组建议下进行更正、更新及引入新的评价[5]。文献检索更新至2020年1月8日，并由专家组成员推荐符合标准的文献。所获得的证据以GRADE方法进行分级，将证据的质量分为四个级别：非常低、低、中等及高[6]。

指南制定专家组根据EtD中汇总的证据，针对每个问题制定了推荐，内容包含证据概要、获益以及不利影响、负担、其他EtD准则和考虑、结论和研究需要这几个方面。最终在证据的质量、不同处理方案的利弊平衡、与决策相关的价值观和偏好几个方面达成共识，获得全部专家组成员的认可[3]。

根据GRADE方法，指南推荐的强度分为“强烈”和“有条件的”的两种。术语“专家组推荐”等同于强烈推荐，“专家组建议”等同于有条件的推荐。

此外，指南中还应用了“良好实践主张”，指的是专家组一致同意有利于患者，但是还没有被广泛认可和使用的干预措施或实践，虽然没有系统性评价的证据支持，仍然视为强烈推荐[3]。

二、临床推荐

2021指南针对涉及6个方面的10个临床问题做出基于循证医学的推荐，主要涉及近年来诊断方面的进展，包括新的实验室检测方法、新的临床亚型的阐述等。既往其他指南中所涉及的基本信息和诊疗原则，此处不再赘述。下面将逐一介绍各问题及相应推荐。

（一）出血评估工具（BAT）

**问题1：**对于疑似VWD的患者，应该采用BAT还是非标准化的临床评估方法进行筛查？

**问题2：**对于疑似VWD患者，尤其是男性及儿童，应用BAT评价为阴性（积分正常）时，是否需要进行血液检测？

**推荐1：**在患有VWD可能性较低的人群中（如初级医疗机构就诊者），专家组推荐应用经过验证的BAT，而不是非标准化的临床评估工具，对患者进行初始筛选以决定哪些患者需要进一步血液检测。（强烈推荐，基于中等质量证据）

**推荐2：**在患有VWD可能性为中等的人群中（如转诊于血液科的就诊者），专家组建议不依赖BAT结果决定是否进行特定的血液检测。（有条件的推荐，基于中等质量证据）

**推荐3：**在患有VWD可能性较高的人群中（如受累患者的第一代亲属），专家组推荐不依赖BAT结果决定是否进行特定的血液检测。（强烈推荐，基于中等质量证据）

**解读：**这三个推荐分别针对不同的VWD患病概率（低、中、高）人群。低VWD患病概率者指在初级医疗机构就诊者，文献显示在这类医疗场景下，实验室检查异常（如APTT延长）的就诊者中有3％为出血性疾病，因此预计VWD患病概率低。中等VWD患病概率者指由于出血史或者实验室筛选实验异常（如APTT延长）而就诊于血液科的人群，针对这种类型连续就诊者的研究显示VWD患病率约为20％。高VWD患病概率者指VWD患者的一级亲属，由于VWD大部分亚型为显性遗传，因此预计这类人群VWD患病概率约为50％（无论是否有出血表现或者实验室检查异常）。

专家组认为应用BAT最主要的获益是能够及时识别出VWD患者，尤其是那些如果不采用BAT评估就可能遗漏的VWD患者，同时降低不必要的血液检测。此外，BAT可以用于评估及记录血液科转诊患者出血症状的严重性，并与其他血液检验一起作为VWD初始诊断方法。BAT也可以为患者和临床医师提供关于出血症状以及干预措施相应的知识。BAT作为筛选手段在成年女性中最为有效，其潜在的不足是可能会遗漏缺乏出血症状的男性及儿童患者。因此在患VWD可能性高的人群中，BAT应该与特定的血液检验共同作为筛选方法。BAT的应用也有助于识别VWD之外的其他出血性疾病，继而通过特定的血液检验鉴别。特定的血液检测包括VWF∶Ag、VWF依赖血小板性活性（如VWF∶GPⅠbM）及FⅧ∶C。可及性方面，公开发表的BAT可以免费获得。ISTH公布了一个专家管理的BAT并为其背书，有多种语言翻译版本，但是目前并没有中文版本。

专家组认为关于BAT应用进一步的研究需要关注不同评分阈值在儿科人群（特别是青少年人群）和男性中的敏感性和特异性。

笔者认为目前国内尚无经过验证的中文版本BAT，个别医院采用自行翻译的ISTH-BAT用以包括VWD在内的出血性疾病评估，使用经验尚少。因此在中国，需要考虑发展适合中国临床实践、经过效度和信度验证的BAT。

（二）VWF血小板依赖性活性检测

**问题3：**对于疑似VWD的患者，应该采用VWF∶RCo检测（自动化和非自动化）还是新的反映VWF血小板结合活性的检测方法（如VWF∶GPⅠbM、VWF∶GPⅠbR）来诊断VWD?

**推荐4：**专家组建议采用新的VWF血小板结合活性检测方法（如VWF∶GPⅠbM、VWF∶GPⅠbR），而不是VWF∶RCo检测（自动化或非自动化）来诊断VWD。（有条件的推荐，基于低质量证据）

良好实践主张：VWF活性应由合适的实验室专业人员来检测。

**解读：**本部分提到的VWF∶GPⅠbR指瑞斯托霉素诱导的VWF与重组野生型GPⅠb片段结合的能力，VWF∶GPⅠbM指VWF与GPⅠbα功能获得性（gain of function）突变体片段自发性结合的能力。两种检测均反映VWF血小板依赖性活性，前者需要瑞斯托霉素介导，后者则无需。

专家组认为与VWR∶RCo相比，新的检测方法变异系数低且重现性高，因此具有一定的获益。另外在某些突变（如发生于非洲人种中的D1472H）时，VWF∶RCo检测值降低，但是并不影响VWF功能，也不增加出血风险，因此VWF∶RCo可能会导致过度诊断。限制这些特定检测方法广泛应用的因素包括这些检测方法的高度专业性（以及操作和解释结果所需的专业知识）、价格、支付机制等。一些检测实验室并不能够提供全部检测项目，因此在临床诊疗中需要选择当地实验室可以提供的检验。

专家组指出，进一步研究重点在于这些项目在不同种族/民族中的结果及对VWF测定的特点以及实验室技术制定国际性指导文件。

VWF血小板依赖性活性检测对于2型VWD的诊断非常重要，但是无论是经典的VWF∶RCo还是新的VWF∶GPⅠbM、VWF∶GPⅠbR，在中国只有极少数医院可以开展，且主要以科研形式开展。目前国内有少数单位临床开展VWF活性（VWF∶Act）检测，其原理是针对VWF的血小板结合位点（GPⅠb受体）的特异性VWF单克隆抗体吸附于乳胶颗粒，与患者血浆中的VWF反应，这种检测方法原理属于直接的抗原-抗体结合，与VWF∶RCo、VWF∶GPⅠbM及VWF∶GPⅠbR检测VWF与GPⅠb（野生型或功能获得性）结合能力的原理有所不同。

（三）VWF水平随着年龄增长正常化

**问题4：**既往诊断为1型VWD，但是现在VWF水平正常的患者，应该重新审议诊断还是取消诊断?

**推荐5：**对于既往确诊为1型VWD但是随着年龄增长VWF水平转为正常的患者，专家组建议重新审议诊断而非取消诊断。（有条件的推荐，基于非常低质量证据）

**解读：**专家组在这个建议下是假设既往1型VWD诊断是准确的。目前已知年龄和其他并发症是可以提高VWF水平的因素，但是VWF水平增高与出血症状之间的关系尚无法确定。

专家组认为重新审议诊断（而非取消诊断）的益处是可以让临床医师评估是否存在其他伴随性出血性疾病（如血小板功能异常），这对于那些诊断1型VWD时并没有进行相关检测的患者尤为重要。专家组认为，VWF正常化程度可能会影响未来出血/手术时的处理决定。对老年患者可能会选择单独使用氨甲环酸，避免使用去氨加压素（DDAVP），以避免心血管并发症和（或）血栓形成。而取消诊断可能会在患者发生出血/手术时无法得到合适的处理，患者也无法得到继续的随访及监测。此外对于某些地区，取消诊断可能导致患者无法被纳入特定的医疗保险。基于以上理由，在做出重新审议或者取消诊断的决定时需要和患者共同决策，需要考虑到患者的价值观及偏向。出血症状比较严重的患者，接受取消诊断的可能性要低于出血轻微的患者。而当患者再次发生出血时，取消VWD的诊断后“原因不明的出血性疾病”可能会给患者带来更大困扰。另外一部分患者为了某些特定工作的要求，则希望取消之前的诊断。

专家组认为VWD的诊断十分复杂，轻度降低的VWF∶Ag和VWF∶RCo或VWF∶GPⅠbM水平并不能确诊VWD；相反，这些指标在正常范围下限时也不能完全排除VWD。数据也未显示VWF水平正常后出血风险降低，因此取消诊断比较困难。进一步的研究焦点在于经过年龄、并发症调整后的VWF水平与出血症状相关性的纵向研究。

笔者认为该问题在中国同样需要关注，尤其对于那些既往VWF∶Ag水平降低，随着年龄增长后恢复正常但仍然有出血表现的患者，取消诊断可能会带来困扰。而另一些患者，可能会因为疾病诊断本身带来困扰，因此倾向于取消诊断。所以，在中国临床实践中，也建议结合患者自身出血表现及意愿做出决定。此外，由于既往针对一些罕见出血性疾病诊断能力的不足，对于此类患者，应重新进行评估以鉴别是否存在VWD以外的其他出血性疾病。

（四）1型VWD

**问题5：**疑似1型VWD的患者初始VWD筛查异常［VWF∶Ag和（或）VWF血小板依赖性活性降低］时，能够确诊的VWF∶Ag和（或）VWF血小板依赖性活性结果的阈值是<0.3 IU/ml还是<0.5 IU/ml?

**推荐6：**专家组推荐无论是否有出血表现，VWF水平<0.3 IU/ml即可确诊为1型VWD，对于有异常出血的患者，VWF水平<0.5 IU/ml即可确诊。（强烈推荐，基于低质量证据）

**解读：**本条推荐中有几点注释需要了解：①VWF水平指的是VWF∶Ag及VWF血小板依赖性活性（如VWF∶GPⅠbM）。②如果当地实验室所确定的VWF水平正常下限是<0.5 IU/ml，应当以当地数据为标准。③虽然O型血VWF水平偏低，但是不需要针ABO血型设定特异的参考范围。④由于VWF是一种急性反应性蛋白，因此需要在患者处于基础状态下进行VWF水平检测。

在以往的指南中，也是将VWF水平<0.3 IU/ml作为确诊1型VWD的标准，但是0.3～0.5 IU/ml之间被定义为低VWF水平，并不作为确诊的阈值。在2021指南中，VWF水平0.3～0.5 IU/ml之间的患者如果有异常出血表现，则可以确诊。专家组认为虽然VWF水平<0.3 IU/ml的患者一般是常染色体显性遗传，但是患者也可能因为不完全外显率以及表达的差异使遗传复杂化。当然VWF水平0.3～0.5 IU/ml伴有出血的患者也可能是由于伴随其他因素导致出血（例如伴随血小板功能异常），使患者出血原因变得复杂，但是专家组认为患者在“确诊”后可以从针对VWD的治疗中获益，虽然也存在治疗相关并发症的风险。

专家组认为虽然仅有低质量证据支持放宽诊断标准可以让患者获益，但是基于以下2点仍然给予强烈推荐：①给予有出血的患者明确诊断以确保他们得到治疗，这点具有很高的价值；②确保诊断标准的国际一致性，并避免有条件的推荐使用中心特定阈值。从以上可以看出，这条推荐是在权衡漏诊和过度诊断后果利弊后的结果，专家组把不漏诊，尤其是那些有出血症状的患者，放在优先位置，以确保患者可接受治疗/预防出血措施。未来的研究重点在于VWF水平0.3～0.6 IU/ml患者的临床特征，包括手术时出血的程度、伴随的出血性疾病的发生率，以及出血表现与家族中1型VWD患者之间的关联。

笔者认为该推荐提高了临床诊断的确定性，避免模糊不清的表述对医师及患者造成的困惑以及增加了相应治疗的合理性，例如确诊患者在使用血浆浓缩FⅧ或冷沉淀及血浆治疗时有明确的依据。

（五）1C型VWD

**问题6：**对于疑似VWF清除率增加引起的1型VWD（1C型VWD），应该用VWF前肽/抗原比值（VWFpp/VWF∶Ag）还是DDAVP试验1 h及4 h检验结果来确定VWF清除率增加？

**推荐7：**专家组建议不要使用VWFpp/VWF∶Ag（VWF前肽与抗原比值），而根据DDAVP输注后1 h及4 h检验结果来证实疑似1C型VWD患者的VWF清除率增加。（条件性推荐，基于低质量证据）

**解读：**在1型VWD中有15％～20％患者是由于VWF清除率增加引起的VWF水平降低，这部分称为1C型VWD。最初完整阐述的病例被命名为Vicenza型VWD，是由于VWF错义突变R1205H所引起的。随后发现此亚型可以通过VWFpp/VWF∶Ag比值鉴别，因为前肽和成熟VWF以1∶1比例储存与内皮细胞的Weibel-Palade小体中，并且在分泌后解离，如果VWF清除加速，VWFpp/VWF∶Ag比值则增加，意味着更短的半衰期。DDAVP试验也可用于鉴别此类患者，输注DDAVP后4 h VWF水平比1 h时（峰值）降低30％时提示VWF清除率增加。

明确诊断1C型VWD对于治疗有重要意义，因为这部分患者可能需要VWF浓缩物治疗/预防出血。DDAVP试验除了协助诊断，也可为这种治疗是否有用提供信息。但是年龄太小或者太大的患者不能进行DDAVP试验，可能会导致低钠血症或者血栓形成等不良反应，VWFpp/VWF∶Ag比值可以用于这部分患者。此外DDAVP试验则需要占用护士及患者时间、诊所空间及检验花费，多次抽血可能会降低患者接受意愿。VWFpp可以简单的通过抽血化验，但是大部分实验室尚未开展此项目。因此虽然专家组认为仅有低质量证据支持DDAVP试验比VWFpp/VWF∶Ag比值诊断1C型VWD获益更佳，但是VWFpp检测能力缺乏时只有DDAVP试验可用于1C型VWD的诊断。

未来研究重点在于研究各种VWFpp/VWF∶Ag阈值的敏感性和特异性、VWFpp的清除率和半衰期，以及相关变量是否恒定，此外还包括包含DDAVP输注后4 h的研究。

VWFpp检测目前在中国可及性很低，仅有少数中心从科研角度检测。而DDAVP的可及性较高，因此利用DDAVP试验鉴别1C型VWD具有很强的合理性及可及性。但是能够掌握关于DDAVP的具体实施方法临床医师不多，因此需要规范化DDAVP试验流程及解读，让临床医师能够正确使用DDAVP试验并进行合理的解读。

（六）2型VWD

**问题7：**对于疑似2型VWD患者，初始VWD筛查异常［低VWF∶Ag和（或）VWF血小板依赖性活性］时，用以确诊2型VWD的VWF血小板依赖性活性/VWF∶Ag比值是<0.5还是更高的阈值<0.7?

**推荐8：**专家组建议应用VWF血小板依赖性活性/VWF∶Ag比值<0.7作为确诊2型VWD（2A、2B、2M）的阈值，反对应用较低的比值<0.5。（条件性推荐，基于非常低质量证据）

**解读：**一些2型VWD患者的VWF∶Ag及VWF血小板依赖性活性可能正常，但是VWF依赖血小板活性/VWF∶Ag比值降低，可以用来诊断2型VWD。

对于这个问题，专家组在讨论时认为假阳性对于临床结局影响不大，因为在诊断2型VWD时通常也会进行其他额外的检测如VWF多聚体分析、VWF∶CB检测以及基因检测，可以进一步确认2型VWD的诊断。相反假阴性对于临床医师及患者影响更大，因为假阴性会导致患者的诊断遗漏，而无法给予合适的处理，VWF活性/VWF∶Ag阈值<0.5时假阴性比例要明显高于<0.7时比例。需要指出的是，该问题是基于初始筛查异常时，因此患者在<0.5阈值下由于假阴性被错误归类为1型VWD的可能性更大，而非漏诊VWD（1型及2型）。

未来需要更多的研究来了解VWF∶RCo在不同种族群体中的变异性。

笔者认为目前VWF血小板依赖性活性检测在中国尚不普及，需要提高国内相关检测的能力，否则将难以正确分型。

**问题8：**对于疑似2A型、2B型或2M型VWD患者而需要进一步检测的患者，应该进行VWF多聚体分析还是VWF∶CB与VWF∶Ag的比值（VWF∶CB/VWF∶Ag）分析?

**推荐9：**专家组建议，对于怀疑为2A型、2B型或2M型需要额外检测的患者，可以采用VWF多聚体分析或VWF∶CB/VWF∶Ag（VWF胶原与抗原结合的比率）诊断2型VWD。（有条件推荐，基于非常低质量证据）

**解读：**VWF∶CB检测并不普及，使用的胶原也不相同：Ⅰ型及Ⅲ型胶原与VWF的A3结构域集合，Ⅳ型胶原与A1结构域结合。大部分实验室用Ⅰ型及Ⅲ型胶原，可以反映高分子量VWF的存在。VWF多聚体分析在技术上有难度，价格远高于VWF∶CB。

正确诊断2A、2B或2M型VWD对预后及家庭咨询非常重要。2型VWD患者通常比1型患者出血更加明显，家族史显示常染色体显性遗传，不存在不完全外显率或表达变异等可以导致1型VWD复杂化的问题。疑似2B型VWD的患者需要接受其他检测以明确诊断，如基因检测。DDAVP治疗对2A及2M型患者可能效果不佳，需要通过DDAVP试验确定疗效。对于2B型VWD患者，DDAVP治疗可能会因为增加血小板结合能力而导致血小板减少，因此在此型中属于相对禁忌。其他的治疗选择在各型之间无特殊区别。

对于已知VWF∶CB异常的VWD患者，需要进一步的研究来评估多聚体诊断检测的准确性。

笔者认为中国目前VWF∶CB及多聚体分析可及性极低，尤其是后者，技术难度大，耗费人力及时间大。基于推荐9，各医院发展上述检验时，可以考虑先从VWF∶CB开始。

**问题9：**对于疑似2A或者2B的VWD患者，应该应用低剂量RIPA还是靶向基因测序来诊断2B型VWD？

**推荐10：**对于疑似2A或2B型需要进一步检测的VWD患者，专家组建议采用靶向基因测序优于低剂量RIPA来诊断2B型VWD。

**解读：**正确诊断2B型VWD与患者预后及治疗相关，因此非常重要。该型患者出血比其他2型及1型VWD更重，出血风险与血小板减少程度相关。此外，该类型属于DDAVP治疗的相对禁忌。

研究发现引起2B型VWD的错义突变位于VWF基因28号外显子，可以通过靶向基因测序检测出错义突变。虽然并不是所有中心都可以进行基因检测，但是邮寄标本到其他实验室比较方便，因此可及性更高，缺点是价格较高。RIPA虽然价格便宜，但也并非所有中心可及，而且该检验需要新鲜标本，因此无法通过样本邮寄送到其他实验室检测。另外，RIPA的检测法没有标准化，各实验室可能使用不同浓度的瑞斯托霉素（0.5、0.25 mg/ml）。

未来需要对RIPA诊断正确性进行研究。

瑞斯托霉素诱导的血小板聚集在一些大型三甲医院可以常规进行，如笔者所在医院常规进行常规浓度及低浓度瑞斯托霉素诱导的血小板聚集试验。自二代测序技术普及以来，多家医院及公司可以提供多种出凝血疾病相关基因的检测，且价格可以接受，通过基因检测方式来确诊是较好选择。

**问题10：**对于疑似2N型VWD而需要进一步检测的患者，应该应用VWF∶FⅧB还是靶向基因测序诊断？

**推荐11：**专家组建议VWF∶FⅧB或靶向基因测序均可以用于诊断2N型VWD。（条件性推荐，基于低质量证据）

**解读：**正确诊断2N型VWD对于治疗、遗传咨询均非常重要。与其他2型及1型VWD不同，2N型是常染色体隐性遗传，这点对于家族内遗传咨询至关重要。因为FⅧ∶C降低，2N型VWD可能被误诊为血友病A，但是二者治疗有所不同：血友病A用FⅧ制剂治疗，2N型需要VWF制剂替代治疗以预防/治疗出血。引起2N型VWD的基因突变一般位于VWF基因第18～20号外显子，其他位点突变也有可能存在但尚未发现。在这些病例中，也需要通过VWF∶FⅧB确认FⅧ与VWF结合能力下降的表型。2N型VWD患者可能为某种突变的纯合子，也可能为2条等位基因的复合杂合突变，也可能为一种突变杂合子同时伴有等位基因的缺失（后代则可能为无出血表现的2N型突变杂合子，或者1型VWD）。因此基因检测相比于VWF∶FⅧB检测能够提供更多遗传咨询的信息。此外，如果疑似患者检测出FⅧ基因突变而非VWF突变，则可确诊为血友病A，需要给予适合血友病A的治疗方式。在实验室检查可及性方面，没有开展基因检测和（或）VWF∶FⅧB检测的中心可以将相应标本（后者为乏血小板血浆）运输至其他实验室检测。因此专家组认为两种方法都可以用来诊断2N型VWD，二者可以在患者的诊断检查中起到互相补充作用。

进一步研究重点在于确定2N型VWD的参考标准。

VWF∶FⅧB目前在中国可及性低。二代测序技术可以在短时间内进行多个相似表型疾病相关基因的测序及分析，可及性比VWF∶FⅧB强。因此笔者认为通过基因检测鉴别2N型VWD，在当下中国更具备可行性。

三、2021指南的更新及不足

既往推荐1型VWD诊断阈值水平为<0.3 IU/ml，对于VWF水平0.3～0.5 IU/ml的患者，NHLBI指南提到如果有出血症状或家族史，则不能排除1型VWD；UKHCDO指南提到这个区间患者如果有出血症状，可以称之为初级止血障碍性出血伴低VWF水平，后者作为出血的风险因素但不诊断为VWD。2021指南的主要原则是避免漏诊误诊，让有出血的患者明确诊断及合适治疗，因此将VWF活性0.3～0.5 IU/ml伴有出血的患者诊断为1型VWD，避免了患者因无法确诊而无法得到合适处理。

2021指南的不足在于部分支持证据为低质量，需要进一步研究来更好回答上面的问题。且对于中国医师及患者来说，2021指南中所涉及到的一些工具和实验室检测方法目前在中国可及性很低，临床应用经验不足。
